# La fasciite nécrosante post opératoire: une complication rare et mortelle

**DOI:** 10.11604/pamj.2016.23.123.8734

**Published:** 2016-03-24

**Authors:** Hassen Ben Ghezala, Najla Feriani

**Affiliations:** 1 Service de Réanimation Médicale, Faculté de Médecine de Tunis, Hôpital de Zaghouan, Zaghouan, Tunisie; 2 Service de Chirurgie Générale à la Faculté de Médecine de Tunis, Hôpital de Zaghouan, Zaghouan, Tunisie

**Keywords:** Fasciite nécrosante, paroi abdominale, post-opératoire, streptocoque, Necrotizing fasciitis, abdominal wall, postoperative, streptococcus

## Abstract

Les complications pariétales post opératoire peuvent être exceptionnellement majeures et graves menaçant le pronostic vital. La fasciite nécrosante est une infection rare de la peau et des tissus sous-cutanés profonds, se propageant le long des fascias et du tissu adipeux. Elle est surtout causée par le streptocoque du groupe A Streptococcus pyogènes mais également par d'autres bactéries telles que Vibrio vulnificus, clostridium perfringens ou Bacteroides fragilis. La fasciite nécrosante est une véritable urgence médicochirurgicale. Nous rapportons dans ce travail une observation très rare d'une gangrène pariétale abdominale survenant chez une patiente de 75 ans au cinquième jour post-opératoire d'un kyste de l'ovaire. L’évolution était marquée par l'installation d'un état de choc septique réfractaire rapidement fatal à J3 de la prise en charge.

## Abstract (en)

Postoperative parietal complications can be exceptionally severe and serious threatening vital prognosis. Necrotizing fasciitis is a rare infection of the skin and deep subcutaneous tissues, spreading along fascia and adipose tissue. It is mainly caused by group A streptococcus (streptococcus pyogenes) but also by other bacteria such as Vibrio vulnificus, Clostridium perfringens or Bacteroides fragilis. Necrotizing fasciitis is a real surgical and medical emergency. We report, in this study, a very rare case of abdominal parietal gangrene occurring in a 75-year-old woman on the fifth day after surgery for an ovarian cyst. Evolution was marked by occurrence of a refractory septic shock with a rapidly fatal course on the third day of management.

## Introduction

Les complications pariétales post opératoires peuvent être exceptionnellement majeures et graves. La fasciite nécrosante est une infection rare de la peau et des tissus sous-cutanés profonds. Elle est surtout causée par le streptocoque du groupe A Streptococcus pyogènes mais également par d'autres bactéries telles que vibrio vulnificus, clostridium perfringens ou bactéroides fragilis. C'est une véritable urgence médicochirurgicale. Nous rapportons dans ce travail le cas fatal d'une fasciite nécrosante de la paroi abdominale survenue chez une femme de 75 ans dans les suites opératoires d'un kyste de l'ovaire.

## Patient et observation

**Figure 1:**
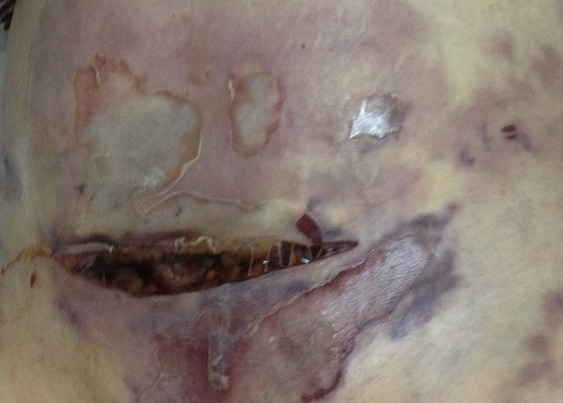
Hématome pariétal et nécrose autour de la cicatrice de Pfannenstiel

**Figure 2:**
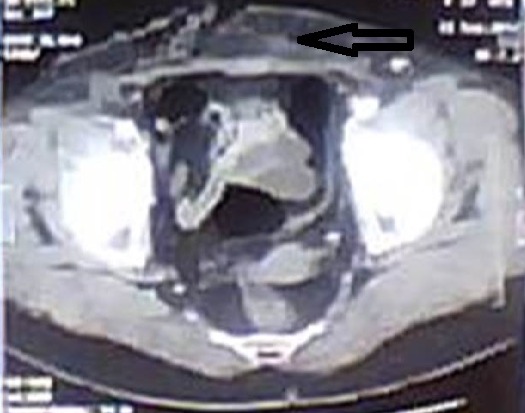
Scanner abdominal montrant une gangrène pariétale infiltrant les tissus sous-cutanées

Il s'agit d'une patiente (Mme D.J) âgée de 75 ans qui a comme antécédents: une obésité androïde grade 2 et un diabète non insulino-dépendant sous antidiabétiques oraux. Elle a été opérée en Juillet 2015 pour ablation d'un kyste de l'ovaire suspect avec des suites opératoires immédiates simples. Cinq jours (5) après son intervention chirurgicale, la patiente consulte le service universitaire d'accueil des urgences de l'hôpital régional de Zaghouan pour douleurs abdominales diffuses et très intenses. L'examen physique aux urgences à l'admission trouve une patiente apyrétique (température à 37^°^5), algique avec un niveau d’échelle visuelle analogique (EVA) à 5 et asthénique. L'examen cardiovasculaire trouve une tachycardie sinusale à 112 battements par minute et une hypotension artérielle à 80\60 mm Hg avec une auscultation cardiopulmonaire normale. L'examen abdominal note une sensibilité abdominale diffuse avec la présence d'un hématome pariétal infiltrant bordant sur une cicatrice récente de Pfannenstiel et atteignant les flancs (Figure 1). On y voit également des plages de nécrose dans le bas ventre au-dessous de la cicatrice. A la biologie, on a trouvé une insuffisance rénale aiguë avec une urée à 15 mmol/l et une créatinémie à 210 µmol/l. L'hémogramme a noté une hyperleucocytose à 19000 éléments/mm^3^ avec une prédominance de polynucléaires neutrophiles (12500 éléments/ mm^3^). La CRP est à 72 mg/l et la procalcitonine à 4 µg/l (Tableau 1). Un scanner abdomino-pelvien fait en urgence était en faveur d'une gangrène pariétale abdominale extensive sans foyer septique intra abdominal (Figure 2). La patiente a été immédiatement admise en unité de soins intensifs. Elle a eu une expansion volémique par du sérum salé isotonique puis elle a nécessité l'intubation, la ventilation mécanique et l'introduction de la noradrénaline comme catécholamine. La patiente a été traitée également par antibiothérapie empirique par voie parentérale à large spectre par Imipénème à la dose de 3 grammes par jour et amikacine à la dose de 1 gramme par jour par voie intraveineuse. Des hémocultures faites à l'admission en unité de soins intensifs ont isolé un streptococcus pyogenes du groupe A sensible à l'amoxicilline. L’évolution était rapidement défavorable avec l'installation d'un état de choc réfractaire à la noradrénaline avec défaillance multi viscérale. La patiente est décédée au troisième jour de la prise en charge en réanimation.

**Tableau 1:** Blan biologique à l'admission en soins intensifs

Bilan Biologique	A l'admission
Urée (mmol/l) / Créatinémie (µmol/l)	15/212
Globules blancs (/mm3)	19000
Polynucléaires neutrophiles (/mm3)	12500
C-Réactive protéine (CRP)	72
Procalcitonine (µg/l)	4.2

## Discussion

La première description des fasciites nécrosantes a été faite par le chirurgien Joseph Jones lors de la guerre civile aux USA en 1871, elles étaient nommées alors «gangrènes hospitalières». Ces fasciites nécrosantes dites de type 2 sont extrêmement rares: 0,3 par 10000 par an [1]. La fasciite nécrosante post opératoire est une infection bactérienne due principalement au streptocoque hémolytique du groupe A et le clostridium perfringens. D'autres bactéries peuvent être mises en cause comme les autres streptocoques, le staphylocoque aureus, le pseudomonas, et les entérobactéries [2–4]. De point de vue physiopathologique, la nécrose est secondaire à la présence de thrombose au niveau de la microcirculation hypodermique due à l'action des toxines et des enzymes bactériennes [2, 5]. Cette nécrose aboutit par la suite à une infection bactérienne par synergie. La fasciite nécrosante est une dermo-hypodermite atteignant la peau, la graisse et se propage le long des fascias avec la vitesse de 2 à 3 cm/heure [6, 7]. Le tableau clinique est dominé par [6]: les signes locaux: (érythème, œdème diffus, phlyctène hémorragique), la nécrose est profonde se manifeste par des taches bleu grisé mal limitées en carte géographique. Il existe également des signes généraux (fièvre, douleur), voir état de choc septique. Le bilan paraclinique est nécessaire pour évaluer la gravité du sepsis et son retentissement multi viscéral. L'hémoculture, les prélèvements bactériologiques sont systématiques [1, 3, 8]. Le scanner est très spécifique pour le diagnostic [9]. Le traitement est médico-chirurgical. La place de l'oxygénothérapie hyperbare est discutée. L’évolution se fait en général vers un sepsis voire un état de choc septique responsable d'une lourde mortalité (30%). Les principaux facteurs de gravité sont essentiellement le diabète, l'obésité, les anti-inflammatoires, l'alcoolisme, les immunosuppresseurs et la rapidité de la prise en charge initiale [9, 10]. Dans notre cas le diabète et l'obésité étaient les facteurs de risque, le tableau clinique est complet, compliqué de choc septique, le prélèvement bactériologique a isolé un streptocoque pygogenes du groupe A et l’évolution était rapidement fatale.

## Conclusion

La fasciite nécrosante est une affection rare, mais gravissime, avec une mortalité atteignant 30%. Le pronostic est amélioré en cas de prise en charge rapide, avec un traitement médico-chirurgical adapté: une exploration chirurgicale qui permet le diagnostic et le traitement et une antibiothérapie adaptée aux germes suspectés. Notre observation montre que malgré une prise en charge adaptée et précoce, c'est une complication particulièrement grave et mortelle.
